# Hybrid Chlorides with Methylhydrazinium Cation: [CH_3_NH_2_NH_2_]CdCl_3_ and Jahn-Teller Distorted [CH_3_NH_2_NH_2_]CuCl_3_

**DOI:** 10.3390/molecules28020473

**Published:** 2023-01-04

**Authors:** Jan A. Zienkiewicz, Dorota A. Kowalska, Dawid Drozdowski, Adam Pikul, Maciej Ptak

**Affiliations:** Institute of Low Temperature and Structure Research, Polish Academy of Sciences, Okólna 2, 50-422 Wrocław, Poland

**Keywords:** methylhydrazinium, organic-inorganic hybrid, chloride, phonon, crystal structure, Jahn–Teller effect

## Abstract

The synthesis, structural, phonon, optical, and magnetic properties of two hybrid organic-inorganic chlorides with monoprotonated methylhydrazinium cations (CH_3_NH_2_NH_2_^+^, MHy^+^), [CH_3_NH_2_NH_2_]CdCl_3_ (MHyCdCl_3_), and [CH_3_NH_2_NH_2_]CuCl_3_ (MHyCuCl_3_), are reported. In contrast to previously reported MHy*M*^II^Cl_3_ (*M*^II^ = Mn^2+^, Ni^2+^, and Co^2+^) analogues, neither compound undergoes phase transitions. The MHyCuCl_3_ has a crystal structure familiar to previous crystals composed of edge-shared 1D chains of the [CuCl_5_N] octahedra. MHyCuCl_3_ crystallizes in monoclinic *P*2_1_/*c* symmetry with MHy^+^ cations directly linked to the Cu^2+^ ions. The MHyCdCl_3_ analogue crystallizes in lower triclinic symmetry with zig-zag chains of the edge-shared [CdCl_6_] octahedra. The absence of phase transitions is investigated and discussed. It is connected with slightly stronger hydrogen bonding between cations and the copper–chloride chains in MHyCuCl_3_ due to the strong Jahn–Teller effect causing the octahedra to elongate, resulting in a better fit of cations in the accessible space between chains. The absence of structural transformation in MHyCdCl_3_ is due to intermolecular hydrogen bonding between two neighboring MHy^+^ cations, which has never been reported for MHy^+^-based hybrid halides. Optical investigations revealed that the bandgaps in Cu^2+^ and Cd^2+^ analogues are 2.62 and 5.57 eV, respectively. Magnetic tests indicated that MHyCuCl_3_ has smeared antiferromagnetic ordering at 4.8 K.

## 1. Introduction

Hybrid organic–inorganic materials (mostly halides) have received a great deal of attention in recent years due to their enormous application potential in the field of high tech industry, particularly as materials for electronic and optoelectronic devices [1,2]. Many of them exhibit controllable optical [3], electric [4], ferroelectric [5,6,7], switchable dielectric [6], and magnetic [8] properties.

Hydrazine is an interesting small inorganic molecule capable of forming an onium or double onium cation that can be incorporated into crystal structures [9,10]. Compounds containing onium hydrazine cations (Hy^+^) are well known and have been the focus of many investigations [9,10]. The degree of methylation of a hydrazine molecule affects its chemical properties and ability to bond in the crystal lattice. Simple organic methylhydrazinium cation (MHy^+^) has just been recognized as an object of interest due to its small enough size to form a three-dimensional (3D) organic–inorganic perovskites [11,12,13,14,15]. Only four organic cations have so far matched the size and shape parameters required to form a 3D perovskite architecture with divalent metal ions. Next to MHy^+^, these include the methylammonium (MA^+^) [16,17,18,19,20], formamidinium (FA^+^) [16,18,20,21,22], and aziridinium (AZ^+^) [23] cations.

There is also a class of halogenide perovskites of larger monovalent metals (*M*^I^ = Na, K, Rb, Cs) that may form a 3D network with the general formula A*M*^I^X_3_·0.5H_2_O, where A stands for bivalent organic cation. In such networks, the bigger dodecahedral gap accommodates larger organic cations, such as dabconium, methyldabconium, 3-aminopyrrolidinium, piperazinium, or methylpiperazinium cations [24,25,26,27].

Recent research has shown that MHy^+^-containing hybrid coordination polymers may also form low-dimensional counterparts, such as layered (2D) [28] or chain (1D) [29,30] architectures. The most intriguing aspect is that the MHy^+^ ligand may interact differently with inorganic metal–ligand subnetworks in the accessible space: (i) organic cation can simply fill the available space and connect with an inorganic network of metal–ligand octahedra [*M*^II^X_6_] through N–H···X (X = oxygen, halide) hydrogen bonds (HBs), as reported in [MHy]*M*^II^(HCOO)_3_ (*M*^II^ = Mn^2+^, Mg^2+^, Fe^2+^, Zn^2+^) [13], [MHy]Mn(H_2_PO_2_)_3_ [31], or in MHyPbI_3_ [29]; (ii) organic cation can be strongly bound with the subnetwork of inorganic octahedra, resulting in additional short N···*M*^II^ contacts, as reported in MHyPbX_3_ (X = Cl^−^, Br^−^) [14,15]; (iii) one of the N atoms can directly be in the first coordination sphere of the metal, forming non-uniform octahedra of the [*M*^II^X_5_N] type, which has so far only been found for the MHy*M*^II^Cl_3_ (*M*^II^ = Mn^2+^, Co^2+^, Ni^2+^) crystals [30]. The third type of rare coordination has also been found for hybrid coordination polymers with Hy^+^, (Hy)_3_MnX_5_ (X = Cl^−^, Br^−^) [32], and 1,1,1-trimethylhydrazinium (Me_3_Hy^+^) [33] cations.

In this study, we synthesized new phases of the hybrid chlorides MHy*M*^II^Cl_3_ that include Cu^2+^ and Cd^2+^ ions. We undertook a comprehensive physicochemical examination to determine why the structural features, including coordination type and interactions of MHy^+^ cations with the metal–chloride framework, of those two analogues vary from other known counterparts. The goal of this work is also to understand why Cu^2+^ and Cd^2+^ analogues do not exhibit phase transitions (PTs) when compared to other members of this family of chlorides.

## 2. Results and Discussion

### 2.1. Structural Properties

**Figure 1:**
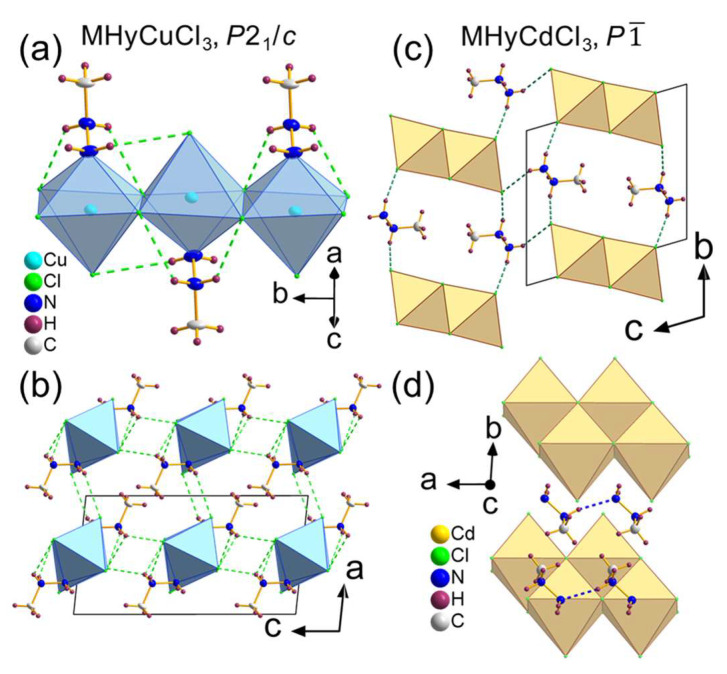
Crystal structures of (**a**,**b**) MHyCuCl_3_ and (**c**,**d**) MHyCdCl_3_; dashed lines represent hydrogen bonds: N–H···Cl (green) and N–H···N (blue).

MHyCuCl_3_ adopted monoclinic *P*2_1_/*c* symmetry. It is yet another example of hybrid MHy*M*^II^Cl_3_ compounds with *M*^II^ = Co^2+^, Ni^2+^, Mn^2+^ reported to date [30], in which the terminal N atom of MHy^+^ is a co-creator of *M*^II^ first coordination sphere. In other words, [CuCl_5_N] octahedra were formed. The octahedra were arranged by edge-sharing, parallel chains propagating along the [010] direction (Figure 1a). The *P*2_1_/*c* phase was isostructural to the low temperature (measured at 100–120 K) phases of Co^2+^, Ni^2+^, and Mn^2+^ analogues [30]. All atoms occupied general positions of *C*_1_ site symmetry. The Cu–Cl distances were 2.2691(10)–2.815(1) Å, while the Cu–N bond length was equal to 2.061(3) Å. The Cu–Cl distances had a much wider range (~0.55 Å) than their Co^2+^ (0.06 Å), Ni^2+^ (0.02 Å), and Mn^2+^ (0.07 Å) counterparts [30]. Indeed, an axial elongation of the octahedra was observed (Figure S1), pointing out the presence of the Jahn–Teller effect, characteristic of Cu^2+^ compounds with octahedral geometries [34]. The MHy^+^ cations were positionally ordered and anchored in the structure by several N–H···Cl HBs (green dashed lines in Figure 1a,b). Both terminal and middle NH_2_ groups interacted with chlorine ion acceptors from neighboring chains, stabilizing the crystal structure in [100] and [001] directions (with donor-acceptor (D···A) distances of 3.426(3) Å and 3.269(3) Å, respectively). The HBs within the chains were also present with D···A distances of 3.157(3)–3.631(3) Å. The intermolecular interactions lead to angular and (combined with Jahn–Teller effect) bond length distortion of the octahedra, as indicated by octahedral angle variance and bond length distortion values of σ^2^ = 20.7 deg^2^ and Δ = 0.1225, respectively. Both values were calculated using the VESTA program [35].

The second newly obtained compound reported herein, i.e., MHyCdCl_3_, crystallized in triclinic, centrosymmetric *P*$\bar{1}$ symmetry. The motif of MHyCdCl_3_ consisted of inorganic [CdCl_3_^−^]_∞_ double chains propagating along the [100] direction, separated by the MHy^+^ cations. All atoms adopted *C*_1_ site symmetry. The chains were composed of edge-sharing octahedra with Cd–Cl distances of 2.523(1)–2.712(1) Å. The MHy^+^ cations were anchored via N–H···Cl (Figure 1c) and N–H···N (Figure 1d) HBs with D···A distances of 3.168(4)–3.384(4) and 2.975(5) Å, respectively. It is worth noting that both the N1 and N2 atoms of MHy^+^ were engaged in creating a 3D network of HBs. An interplay between inorganic and organic constituents affected the octahedra symmetry, expressed in σ^2^ and Δ values of 17.8 deg^2^ and 0.021, respectively. Analogous atomic alignment, i.e., with 1D edge-sharing double chains, was reported for MHyPbI_3_ [29]. Higher symmetry of MHyPbI_3_ (monoclinic, *P*2_1_/*c*) is associated with the presence of larger metal cations and halide anions, which leads to enlarged interatomic distances and, therefore, weaker HBs and less distorted octahedra (σ^2^ = 6.32 deg^2^). Analogous alignment was also reported for [C_3_H_7_N_2_S]CdCl_3_, where C_3_H_7_N_2_S^+^ is the 2-amino-4,5-dihydro-3*H*^+^-1,3-thiazolium cation [36].

**Figure 2:**
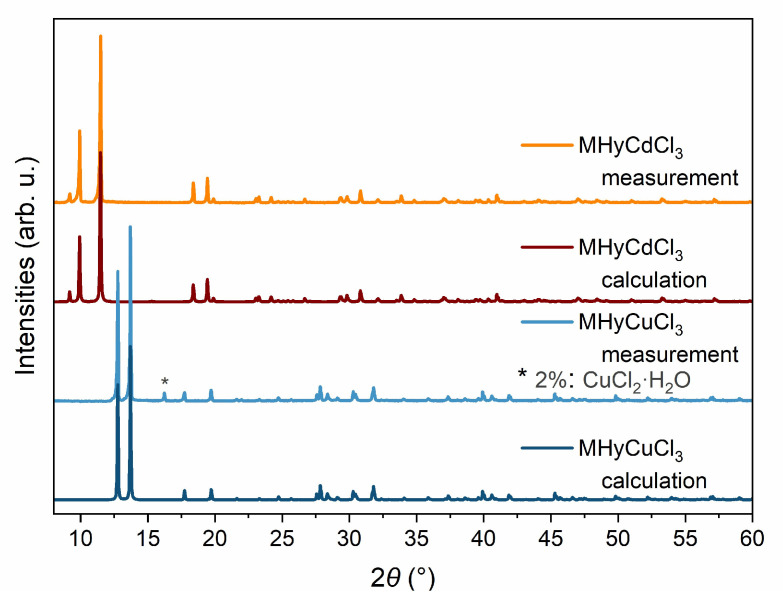
The comparison of the experimental and calculated using Pawley method PXRD patterns for MHyCdCl_3_ and MHyCuCl_3_ samples showing the quality of phase purity; * the additional phase is marked with an asterisk.

The phase purity of the MHyCdCl_3_ bulk sample was confirmed by a good match of its PXRD pattern (*R*_exp_ = 1.70, *R*_prof_ = 6.26, *wR*_prof_ = 11.22, *GOF* = 6.6) with the simulated one based on the single crystal structure (Figure 2). The measured PXRD pattern of the MHyCuCl_3_ bulk sample was also in good agreement (*R*_exp_ = 1.52, *R*_prof_ = 5.44, *wR*_prof_ = 9.85, *GOF* = 6.5) with the calculated one based on the single-crystal data. The Pawley refinement method was used to obtain the fitted profiles. The PXRD analysis results also revealed the negligible presence of another phase (CuCl_2_·H_2_O) in an amount of about 2%; the peak from the additional phase is marked with an asterisk in Figure 2.

### 2.2. Phonon Properties

Table S1 defines and lists the 24 internal (13A′ + 11A″) and 6 external (3A′ + 3A″) vibrational modes of the free MHy^+^ ion with *C*_s_ symmetry. The presence of two MHy^+^ ions in the primitive cell doubles the number of modes corresponding to MHy^+^ with the factor group symmetry *C*_i_ in the triclinic MHyCdCl_3_ crystal with *Z* = 2. As a result, the internal and external modes are increased to 48 (24A_g_ + 24A_u_) and 12 (6A_g_ + 6A_u_), respectively. Since the number of modes corresponding to MHy^+^ cations with the *C*_2h_ symmetry is increased by 4 times in the monoclinic MHyCuCl_3_ crystal with *Z* = 4, the number of modes corresponding to MHy^+^ cations with the *C*_2h_ symmetry is 96 (24A_g_ + 24A_u_ + 24B_g_ + 24B_u_) and 24 (6A_g_ + 6A_u_ + 6B_g_ + 6B_u_), respectively. Similar considerations apply to metal cations *M*^II^ and chloride ligands Cl^−^, which have 6 (3A_g_ + 3A_u_) and 18 (9A_g_ + 9A_u_) modes in the triclinic MHyCdCl_3_ crystal, respectively, and 12 (3A_g_ + 3A_u_ + 3B_g_ + 3B_u_) and 36 (9A_g_ + 9A_u_ + 9B_g_ + 9B_u_) modes in the monoclinic MHyCuCl_3_ crystal.

**Figure 3:**
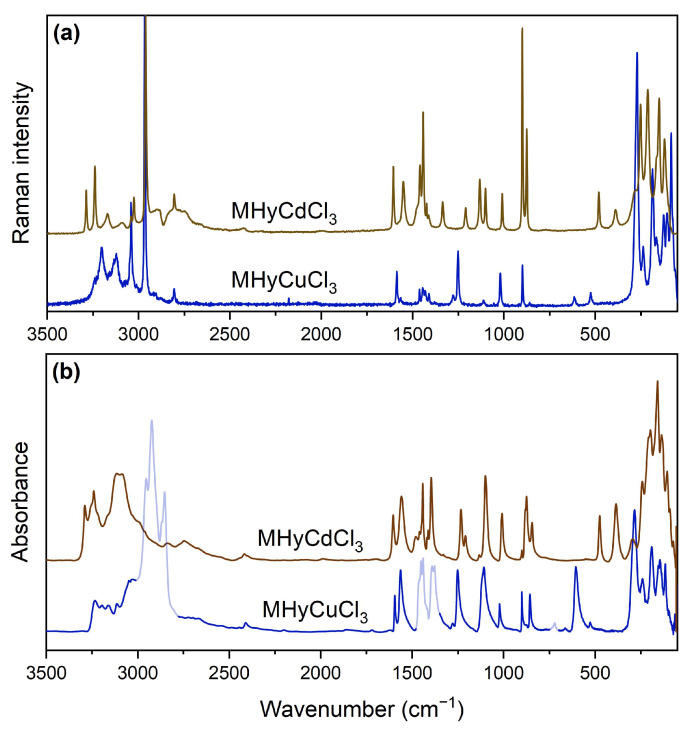
Raman (**a**) and IR (**b**) spectra of MHy*M*^II^Cl_3_ (*M*^II^ = Cd^2+^, Cu^2+^); transparent sections of the MHyCuCl_3_ IR spectrum on panel (**b**) indicate the absorption ranges of the medium used (nujol).

To summarize, the total number of expected vibrational modes for MHyCdCl_3_ is 84 (42A_g_ + 42A_u_), which includes 81 optical (42A_g_ + 39A_u_) and 3 acoustic (3A_u_), and 168 (42A_g_ + 42A_u_ + 42B_g_ + 42B_u_) for MHyCuCl_3_, which includes 165 optical (42A_g_ + 41A_u_ + 42B_g_ + 40B_u_) and 3 acoustic. Because *g*-type modes are only Raman-active and *u*-type modes are only IR (infrared)-active, the number of expected bands in the Raman (IR) spectrum of MHyCdCl_3_ is 42 (39). These values are 84 and 81 for the MHyCuCl_3_ analogue, respectively. The number of observed bands in the room-temperature (RT) spectra is lower than expected (Figure 3, Table S2). This effect is caused by the overlapping of closely spaced bands caused by the low factor group (Davydov) splitting.

The proposed assignment, presented in Table S2, is based on a comparison with literature sources, including MHy*M*^II^Cl_3_ (*M*^II^ = Co^2+^, Ni^2+^, Mn^2+^) [30], 3D and 2D lead halides comprising the MHy^+^ cation [12,29,37], MHyMn(H_2_PO_2_)_3_ [31], and MHy*M*^II^(HCOO)_3_ (*M*^II^ = Mg^2+^, Fe^2+^, Mn^2+^, and Zn^2+^) [13], as well as density functional calculations (DFT) calculations performed for the MHy^+^ ion [31]. The majority of IR and Raman bands corresponding to the internal vibrations of MHy^+^ are observed in typical ranges as reported in the literature.

The IR and Raman spectra of MHyCuCl_3_ are qualitatively very similar to those of MHy*M*^II^Cl_3_ (*M*^II^ = Co^2+^, Ni^2+^, Mn^2+^) [30]. The differences involving wavenumber up- or downshifts reaching a few cm^−1^ for MHyCuCl_3_ are mainly due to the higher mass, different ionic radius, the strength of HBs, and Jahn–Teller effect activity of Cu^2+^ ions in an octahedral configuration. The most pronounced variations were observed for the so-called MHy^+^-cage mode, which can be correlated with the parameter defined as the space available for cation per formula unit *V*_Z_ [30]. This mode was described as a torsional mode with a strong sensitivity to ligand type due to coupling with the inorganic cage via HBs bonds [37], which is similar to the behavior of the MA^+^-cage mode, which has been initially reported for the MAPbX_3_ (X = Br^−^, I^−^) hybrids [38]. In our previous paper concerning MHy*M*^II^Cl_3_ (*M*^II^ = Co^2+^, Ni^2+^, Mn^2+^), the MHy^+^-cage mode clearly correlates to the ionic radius of *M*^II^ and metal electronegativity, i.e., it was observed as an IR band at 515 cm^−1^ for MHyMnCl_3_, 563 cm^−1^ for MHyCoCl_3_, and at 595 cm^−1^ for MHyNiCl_3_ [30]. Taking into account the IR and Raman spectra of MHyCdCl_3_ and MHyCuCl_3_, we assigned the MHy^+^-cage mode to IR bands at 386 and 606 cm^−1^, as well as Raman bands at 389 and 614 cm^−1^, respectively (see Table S2). The correlation of these values with the ionic radius is higher for MHyCuCl_3_ and much lower for MHyCdCl_3_. This is not surprising considering that MHy^+^ is not a direct ligand for the metal cation in the MHy*M*^II^Cl_3_ series. As a result, the torsional vibrational energy is predicted to differ.

Another interesting feature that can be analyzed using vibrational spectroscopy is the strength of HBs. The positions of IR and Raman bands corresponding to stretching vibrations of both NH_2_ and NH_2_^+^ groups for the MHyCuCl_3_ analogue have very similar energies in comparison to the previously studied MHy*M*^II^Cl_3_ (*M*^II^ = Co^2+^, Ni^2+^, Mn^2+^) series. However, for MHyCuCl_3_, most of the bands observed above 3000 cm^−1^ have the lowest positions among these four isostructural analogues. It proves our SCXRD analysis, showing that the MHyCuCl_3_ analogue creates the shortest D···A contacts and thus forms the strongest hydrogen bonds. 

As mentioned before, the vibrational spectra of MHyCdCl_3_ differ most strongly in this region, suggesting that the HB network has different properties. Crystallographic data showed that the MHy^+^ cations do not enter the first coordination sphere of Cd^2+^ ions, therefore, they have more freedom in the metal–chloride framework. This leads to the formation of direct, short, and strong N–H···N HBs between MHy^+^ cations, which has never been reported for an MHy^+^ cation so far. We think that these stronger contacts are responsible for the formation of the broad bands observed in spectra above 2750 cm^−1^, which are absent for other representatives of the MHy*M*^II^Cl_3_ group.

### 2.3. Optical Properties

**Figure 4:**
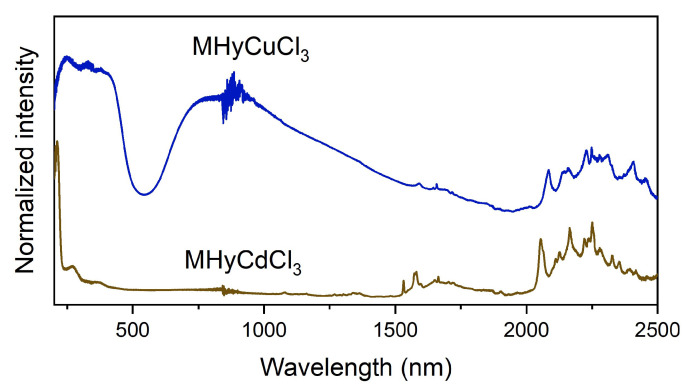
Diffuse reflectance spectra of MHy*M*^II^Cl_3_ (*M*^II^ = Cd^2+^, Cu^2+^).

The diffuse reflectance spectra of MHy*M*^II^Cl_3_ (*M*^II^ = Cd^2+^, Cu^2+^) are shown in Figure 4. In the low-wavelength range, MHyCdCl_3_ exhibits an intense absorption band with a maximum at 212 nm and a weaker band at 275 nm, similar to FACd(H_2_PO_2_)_3_ (FA^+^ = formamidinium) [39] and [TPrA]Cd(dca)_3_ (TPrA^+^ = tetrapropylammonium, dca^−^ = dicyanamide) [40]. MHyCuCl_3_ has a much wider low-wavenumber band, with maxima at 249, 330, 379, and 410 nm previously assigned to the ligand-to-metal charge transfer from Cl^−^ ligand to Cu^2+^ centers for (C_5_H_14_N_2_)[CuCl_4_] (C_5_H_14_N_2_^2+^ = diprotonated 1,4-diazacycloheptane), (N(CH_3_)_4_)CdX_3_:Cu^2+^ (N(CH_3_)_4_^+^ =tetramethylammonium, X = Cl^−^, Br^−^), and MA_2_CuCl_x_Br_4-x_ (MA^+^ = methylammonium) [41,42,43]. The diffuse reflectance spectrum of MHyCuCl_3_ also has a very broad absorption band with a maximum of about 850 nm, which is caused by Cu^2+^ *d*-*d* transitions in the *D*_4h_ coordination centers [41,43,44]. The presence of this band for MHyCuCl_3_, which is composed of CuCl_6_ octahedra, indicates a strong Jahn–Teller effect causing octahedra elongation, as confirmed by SCXRD studies (see Figure S1).

Both spectra show the presence of several bands above 1500 nm that corresponds to MHy^+^ overtones and combinations of vibrational modes.

The diffuse reflectance spectra of MHy*M*^II^Cl_3_ (*M*^II^ = Cd^2+^, Cu^2+^) were further recalculated to determine the bandgap energy using the Kubelka–Munk function *F*(*R*) = (1 − *R*)^2^/2*R*, where *R* is the reflectance. The results are presented in Figure S2, and the obtained bandgap energies are 2.62 and 5.57 eV for MHyCuCl_3_ and MHyCdCl_3_, respectively. The obtained values are within the ranges reported previously for (C_5_H_14_N_2_)[CuCl_4_] (2.56 eV) [41], FACd(H_2_PO_2_)_3_ (5.42 eV) [39], and [TPrA]Cd(dca)_3_ (5.02 eV) [40].

### 2.4. Magnetic Properties

**Figure 5:**
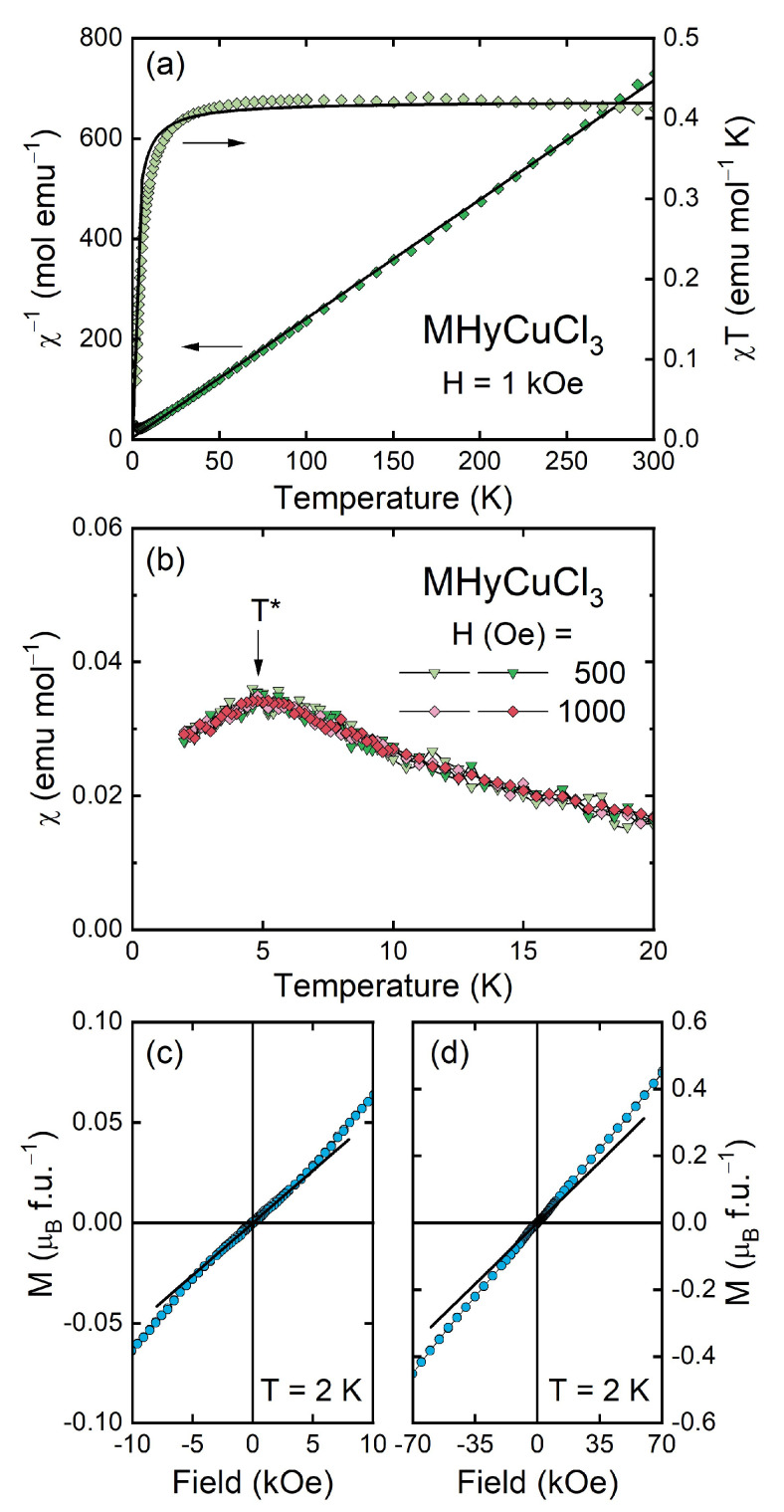
(**a**) *χ*^−1^(*T*) and *χT*(*T*) (left and right axis, respectively) of MHyCuCl_3_ plotted with the fitted Curie–Weiss formula (solid curves). (**b**) *χ*(*T*) measured in the ZFC and FC regimes (bright and dark symbols, respectively); the arrow marks a characteristic temperature T*. (**c**,**d**) *M* vs. *H* plotted in two different field ranges; solid lines show linear *M*(*H*) dependence in low fields.

Figure 5 summarizes the results of the magnetic property measurements carried out for MHyCuCl_3_, which are very similar to those obtained for MHyMnCl_3_ [30]. As can be inferred from panel (a), the compound with copper exhibited paramagnetic behavior in almost the entire temperature range studied, and its magnetic susceptibility *χ*(*T*) could be described by the Curie–Weiss law *χ*(*T*) = *C*/(*T* − *θ*_p_) down to a few kelvins with the least-squares fitting parameters *C* = 0.42(2) emu mol^−1^ K (Curie constant) and *θ*_p_ = −1.7(4) K (Curie–Weiss temperature); see the thick solid line in Figure 5a. The effective magnetic moment *μ*_eff_ derived from the Curie constant *C* was about 1.83(1) μ_B_, which was lower than *μ*_eff_ = 3.55 μ_B_ expected for a free Cu^2+^ ion with the 3d^9^ electron configuration (i.e., for *S* = 1/2, *L* = 2, *J* = 5/2, *g*_J_ = 1.2) and only slightly higher than the spin-only magnetic moment of 1.73 μ_B_ (i.e., calculated for *S* = 1/2, *L* = 0, *J* = 1/2, *g*_J_ = 2), suggesting a non-negligible, yet still very small, orbital contribution to *μ*_eff_. The estimated value of the magnetic moment was, in turn, very close to the averaged experimental value of *μ*_eff_ reported for paramagnetic salts containing non-interacting Cu^2+^ ions, i.e., 1.83 μ_B_. Moreover, the fitted value of *C* was nearly the same as the RT value of the product *χT*, i.e., 0.41 emu mol^−1^ K, and the negative sign of *θ*_p_, which indicated the presence of antiferromagnetic correlations in MHyCuCl_3_, was in full agreement with the concave shape of the *χT*(*T*) curve observed down to the lowest temperature studied (see Figure 5a, right axis).

At low temperatures, MHyCuCl_3_ exhibited a broad anomaly in the temperature variation of the magnetic susceptibility with a maximum at T* = 4.8 K (Figure 5b), being very similar in shape to that found in its counterpart with Mn^2+^, yet much smaller [30]. In addition, here, *χ*(*T*) was independent of the magnetic field *H* (at least in low fields) and showed no difference between the curves taken in the ZFC and FC regimes, which suggests antiferromagnetic ordering. The antiferromagnetic-like character of the observed anomaly was confirmed by the linear field dependence of the magnetization (Figure 5c,d), exhibiting only a small change in its slope of about 3 kOe. In the highest field applied (70 kOe), magnetization *M* is far from any saturation and achieves a value corresponding to only 0.45 μ_B_, which is much smaller than *μ*_ord_ = 3 μ_B_ and 1 μ_B_ expected for full and spin-only magnetic moment of Cu^2+^, respectively.

### 2.5. Effect of Cd^2+^ and Cu^2+^ on the Properties

Cd^2+^ and Cu^2+^ coordination chemistry significantly differs from Ni^2+^, Co^2+^, and Mn^2+^, which has a significant influence on the structures of MHyCdCl_3_ and MHyCuCl_3_ in comparison to previously described compounds. To begin, unlike the other metals discussed, Cd^2+^ has a *d*^10^ close-shell electronic configuration. Additionally, according to Shannon [45], the ionic radius of Cd^2+^ is greater (95 pm) than that of Ni^2+^, Co^2+^, Mn^2+^, and Cu^2+^ (69–83 pm). Because of these features, the metal cation in MHyCdCl_3_ is coordinated by six chloride anions, and the MHy^+^ cation is excluded from the first coordination sphere. MHyCdCl_3_ has a triclinic structure, with the MHy^+^ cations occupying the voids in the metal–chloride framework. Furthermore, MHy^+^ cations are stacked in chains that are linked by stronger intermolecular HB.

The distinct behavior of Cd^2+^ and Cu^2+^ analogues has previously been observed for other hybrids. Among the Am*M*^II^(HCOO)_3_ (Am^+^ = NH_4_^+^; *M*^II^ = Cd^2+^, Co^2+^, Cu^2+^, Fe^2+^, Mg^2+^, Mn^2+^, Ni^2+^, and Zn^2+^) formates [46], only AmCd(HCOO)_3_ and AmCu(HCOO)_3_ members adopt the orthorhombic symmetry, which is lower compared to other hexagonal family members. Furthermore, as the sole representative, the AmCd(HCOO)_3_ crystal exhibits no PT and adopts a perovskite-like topology [47] in contrast to other members, which have chiral-like crystal architecture.

A similar outcome effect has been observed for [DMA]*M*^II^(HCOO)_3_ (DMA^+^ = dimethylammonium; *M*^II^ = Cd^2+^, Co^2+^, Cu^2+^, Fe^2+^, Mg^2+^, Mn^2+^, Ni^2+^, and Zn^2+^) formates [46], where only [DMA]Cu(HCOO)_3_ and [DMA]Cd(HCOO)_3_ do not undergo PTs. However, the structural symmetry of [DMA]Cd(HCOO)_3_ is hexagonal in this example, as it is for all members except for [DMA]Cu(HCOO)_3_, which crystallizes in the lower orthorhombic phase. In this case, the lack of PT has been attributed to the large size of the cavity occupied by DMA^+^ and weak HB contacts between cations and the cadmium-formate framework [48].

To determine the reason for the absence of PTs in the MHyCdCl_3_ and MHyCuCl_3_, we compared the estimated structural parameters presented in Table 1. As one can see, the tolerance factor (TF) is insufficient to explain this phenomenon, as the values for MHyCdCl_3_ and MHyCuCl_3_ are comparable to those reported for MHy*M*^II^Cl_3_ (*M*^II^ = Mn^2+^, Co^2+^, Ni^2+^) [30]. A detailed examination of σ^2^ and Δ, which characterize the framework flexibility, reveals that the Jahn–Teller effect prevents the emergence of PT. This elongation also causes the shortest Cu–Cu distances between octahedral chains propagating along the [100] and [001] directions to be 6.988(1) and 6.505(1) Å, respectively. The *M*^II^–*M*^II^ distances or MHy*M*^II^Cl_3_ (*M*^II^ = Mn^2+^, Co^2+^, Ni^2+^) are comparable along the [001] direction (6.489(1)–6.580(1) Å) but much longer along the [100] direction (7.156(1)–7.338(1) Å) [30]. The closest Cu–Cu distances (3.601(1)–3.661(1) Å) along the chain are comparable or slightly higher than the *M*^II^–*M*^II^ distances reported for *M*^II^ = Mn^2+^, Co^2+^, Ni^2+^, which ranged from 3.455(1) to 3.630(1) Å [30]. Furthermore, among the other members, the Cu–N bond is the shortest (2.061(3) Å) and contributes the most covalent bonding (2.116(3), 2.194(2), and 2.365(2) Å for Ni^2+^, Co^2+^, and Mn^2+^, respectively). The substantially elongated [CuCl_5_N] octahedra change the available space for MHy^+^ cations and allow them to fit better in the accessible void, resulting in a slightly stronger network of HBs. This alignment in the metal-halide network might possibly be due to the ordered state of the MHy^+^ cations, which has been reported for MHy*M*^II^Cl_3_ (*M*^II^ = Mn^2+^, Co^2+^, Ni^2+^) in the low-temperature (LT) phase [30].

The inability of Cd^2+^ ions to bind MHy^+^ also makes these cations better fit into the existing spaces in the network and can get close enough to form a HB between two adjacent cations. The existence of this unique contact, which is stronger than the HBs normally formed between organic cations and ligands, efficiently prevents cation disorder and allows the crystal to adopt lower triclinic symmetry with double edge-connected zig-zag octahedral chains, which is impossible for Ni^2+^, Mn^2+^, and Co^2+^ transition metal cations.

## 3. Materials and Methods

### 3.1. Synthesis

Methylhydrazine (98%, Sigma-Aldrich, Saint Louis, MS, USA), hydrochloric acid (35–38%, Avantor Performance Materials, Gliwice, Poland), cadmium(II) (98%, Sigma-Aldrich, Saint Louis, MS, USA), and copper(II) (98%, Sigma-Aldrich, Saint Louis, MS, USA) chlorides were obtained commercially and used without additional purification.

In order to grow MHy*M*^II^Cl_3_ (*M*^II^ = Cu^2+^, Cd^2+^) crystals, 1 mmol of *M*^II^Cl_2_ was digested in hydrochloric acid. The solution was then dropwise treated with methylhydrazine (1.5 mmol, 0.2 mL). The resulting mixture was left undisturbed at RT in order to slowly evaporate the solvent. After 7–30 days, the colorless and green crystals of MHyCdCl_3_ and MHyCuCl_3_, respectively, were harvested from the solution and air-dried.

### 3.2. Single-Crystal and Powder X-ray Diffraction

Single-crystal X-ray diffraction (SCXRD) experiments were carried out with MoKα radiation using an Xcalibur four-circle diffractometer (Oxford Diffraction, Abingdon, UK), an Atlas CCD detector, and graphite-monochromated MoKα radiation. Absorption was corrected by multi-scan methods using CrysAlis PRO 1.171.39.46 (Rigaku Oxford Diffraction, 2018, Tokyo, Japan). Empirical absorption correction using spherical harmonics, implemented in the SCALE3 ABSPACK scaling algorithm, was applied. Hydrogen atoms were initially placed based on the local geometry and refined using a riding model. The crystal structure was solved in Olex2 1.5 [49] using SHELXT-2014/4 [50] and refined with SHELXL-2018/3 [51]. The unit cell of MHyCuCl_3_ (monoclinic, *P*2_1_/*c* with *a* = 6.9879(5) Å, *b* = 7.2032(5) Å, *c* = 13.0105(9) Å, *β* = 96.022(7)°, *V* = 651.3(1) Å^3^, *Z* = 4) was chosen with respect to the analysis of diffraction pattern and systematic extinction rules (Figure S3). MHyCdCl_3_ (triclinic, *P*$\bar{1}$ with *a* = 3.8660(1) Å, *b* = 9.3519(9) Å, *c* = 10.0790(3) Å, *α* = 106.146(6)°, *β* = 90.080(3)°, *γ* = 93.734(5)°, *V* = 349.21(4) Å^3^, *Z* = 2) was treated as a two-domain non-merohedral twin with twin fraction (BASF) equal to 0.5358(11). Experimental details and selected geometric parameters are presented in Tables S3–S5. The main components of the crystal structures of both compounds are presented in Figure S4. The CIF files of reported structures can be found in the CCDC Database with deposition numbers 2047529 for MHyCdCl_3_ and 2047531 for MHyCuCl_3_.

Powder X-ray diffraction (PXRD) experiments were performed in the reflection mode on a PANalytical X’Pert diffractometer (Almelo, The Netherlands) equipped with a a PIXcel solid-state linear detector using Ni filtered CuKα radiation (*λ* = 1.54184 Å). The X-ray diffraction patterns were generated at 30 mA and 40 kV. For the processing of the PXRD data, the program X’Pert High Score Plus (PANalytical, Almelo, The Netherlands) was involved [52].

### 3.3. Spectroscopic Measurements

IR spectra in the range of 4000–400 cm^−1^ (mid-IR) were measured using a Nicolet iS50 infrared spectrometer (Waltham, MA, USA) using a KBr pellet for MHyCdCl_3_ and a nujol suspension for MHyCuCl_3_ due to the reactivity of the compound with KBr. The far-IR spectra in the 400–50 cm^−1^ were measured as a nujol suspension on the polyethylene plate for both compounds. The spectral resolution was set to 2 cm^−1^.

Raman spectra in the 3500–50 cm^−1^ range with 2 cm^−1^ resolution were measured using a Bruker FT MultiRAM spectrometer (Billerica, MA, USA) equipped with the YAG:Nd laser operating at 1064 nm.

The absorption spectra in the back-scattering mode in the UV-VIS range were measured using an Agilent Cary 5000 spectrophotometer (Santa Clara, CA, USA) equipped with a PryingMantis™ diffuse reflectance accessory.

### 3.4. Magnetic Measurements

Magnetization of randomly oriented single crystals of MHyCuCl_3_ was measured using a commercial Quantum Design MPMS XL magnetometer (San Diego, CA, USA) from RT down to 2 K and in applied magnetic fields up to 70 kOe. The diamagnetic background coming from a sample holder was found to be weak and negligible in comparison to the signal coming from the samples; hence its subtraction was omitted. Moreover, no diamagnetization corrections were made to the data reported here.

## 4. Conclusions

The MHy*M*^II^Cl_3_ (*M*^II^ = Cd^2+^, Cu^2+^) hybrid organic–inorganic compounds were synthesized using a conventional technique of crystallization from solution by gradual evaporation. The MHyCuCl_3_ analogue crystallizes in the monoclinic *P*2_1_/*c* symmetry, according to the single-crystal X-ray diffraction measurement. The structure is similar to previously reported *M*^II^ = Mn^2+^, Ni^2+^, and Co^2+^ structures in that it is made up of edge-sharing 1D chains of the [CuCl_5_N] octahedra running in the [010] direction. MHy^+^ cations in MHyCuCl_3_ are effectively coordinated by metal ions, as in the three analogues mentioned. MHyCuCl_3,_ as a single representative, on the other hand, experiences no PT, and the MHy^+^ cations are ordered at RT. The absence of PTs was attributed to the significant Jahn–Teller effect, which was supported by single-crystal X-ray diffraction and diffuse reflectance measurements. The elongation of the [CuCl_5_N] octahedra results in a better fit of organic cations in the accessible space as well as a considerably stronger network of HBs that prevent the disorder. Magnetic measurements revealed that MHyCuCl_3_ exhibits only smeared antiferromagnetic ordering at roughly 4.8 K and no ferromagnetic correlations up to the highest field investigated. Optical studies confirmed a strong Jahn–Teller distortion and revealed a bandgap of 2.62 eV for this material.

The cadmium analogue differs significantly from the other representatives in the MHy*M*^II^Cl_3_ class of hybrids. The MHyCdCl_3_ compound crystallizes in triclinic $P\bar{1}$ symmetry, and its crystals are comprised of double zig-zag chains that propagate along the [100] direction. The MHy^+^ cations are ordered at RT, and the MHyCdCl_3_ crystal, like MHyCuCl_3_, does not experience PTs. In this case, the absence of structural transition was attributed to the presence of a unique HB contact between two neighboring MHy^+^ cations, which inhibits cation disorder. This type of contact is possible because, uniquely in this case, the organic cations are not included in the first coordination sphere of the Cd^2+^ ion. According to optical investigations, MHyCdCl_3_ has a bandgap energy of 5.57 eV.

## Figures and Tables

**Table 1:** Comparison of tolerance factors (TFs), octahedral angle variance σ^2^, and bond length distortion Δ for MHy*M*^II^Cl_3_.

*M* ^II^	TF	Δ	σ^2^ (deg^2^)
Mn^2+^ [30]	1.16	0.0240	32.093
Co^2+^ [30]	1.23	0.0311	18.368
Ni^2+^ [30]	1.26	0.0350	9.569
Cu^2+^	1.24	0.1225	20.661
Cd^2+^	1.14	0.0210	17.839

## Data Availability

Data available on request.

## Supplementary Materials

The following supporting information can be downloaded at: https://www.mdpi.com/article/10.3390/molecules28020473/s1, Figure S1: Single [CuCl_5_N] octahedron labeled with atoms and bond lengths in Å; elongated Pb-Cl distances (marked in red and bold) indicate the Jahn–Teller effect; Figure S2: The energy band gap estimation for MHy*M*^II^Cl_3_ (Cd, Cu) crystals using the Kubelka–Munk method; Figure S3: Reciprocal space reconstruction of (a) the *h*0*l* and (b) the *h*1*l* layers in MHyCuCl_3_; Figure S4: The main components of the crystal structures of (a) MHyCuCl_3,_ and (b) MHyCdCl_3_ with vibrational ellipsoids at the 50% probability level; Table S1: Factor group analysis for MHyCdCl_3_ and MHyCuCl_3_ crystals; Table S2: Assignment of IR and Raman bands for MHyCdCl_3_ and MHyCuCl_3_; Table S3: Experimental and refinement details of MHyCdCl_3_ and MHyCuCl_3_; Table S4: Selected geometric parameters of MHyCdCl_3_ and MHyCuCl_3_ (Å, °); Table S5: Selected hydrogen bond parameters of MHyCdCl_3_ and MHyCuCl_3_.

## Sample Availability

Samples of the compounds MHyCdCl_3_ and MHyCuCl_3_ are available from the authors.
