# Supplementary material for: Hybrid Chlorides with Methylhydrazinium Cation: [CH3NH2NH2]CdCl3 and Jahn-Teller Distorted [CH3NH2NH2]CuCl3
Source: Molecules. 2023 Jan 4;28(2):473. doi: 10.3390/molecules28020473 (PMC9865361; doi:10.3390/molecules28020473)
Supplement: Supplementary file 1 [file molecules-28-00473-s001.zip › molecules-2106176-supplementary.pdf]

Supporting Information for

Hybrid chlorides with methylhydrazinium cation: $[CH_3NH_2NH_2]CdCl_3$ and Jahn-Teller distorted $[CH_3NH_2NH_2]CuCl_3$

J. A. Zienkiewicz,* D. A. Kowalska, D. Drozdowski, A. Pikul and M. Ptak*

Institute of Low Temperature and Structure Research, Polish Academy of Sciences, Okólna 2, 50-422 Wrocław, Poland

Correspondence: j.zienkiewicz@intibs.pl (J.A.Z.), m.ptak@intibs.pl (M.P.)

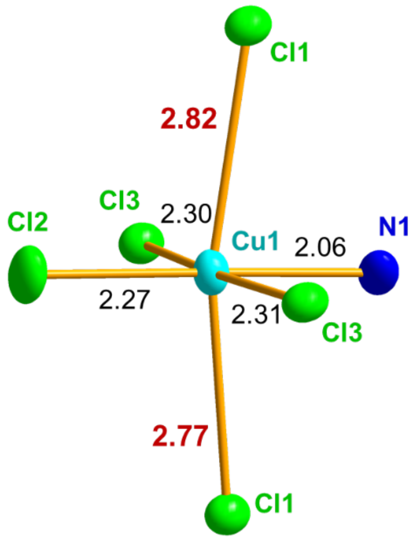

Figure S1. Single $[CuCl_5N]$ octahedron labeled with atoms and bond lengths in Å; elongated Cu–Cl distances (marked in red and bold) indicate the Jahn-Teller effect.

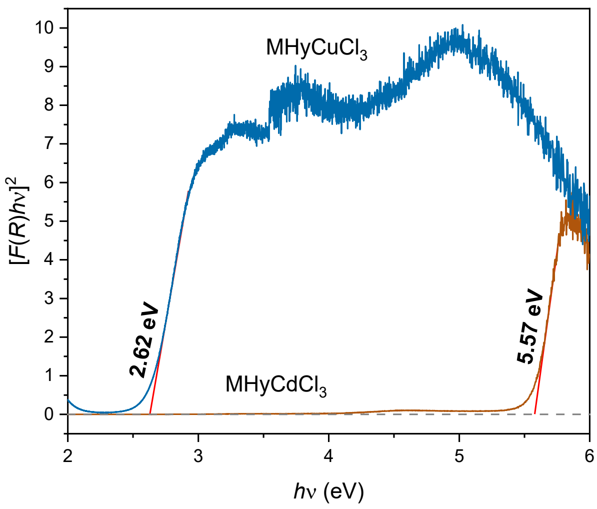

Figure S2. The energy band gap estimation for MHy$M^{II}Cl_3$ (M^{II}=Cd^{2+}, Cu^{2+}) crystals using the Kubelka-Munk method.

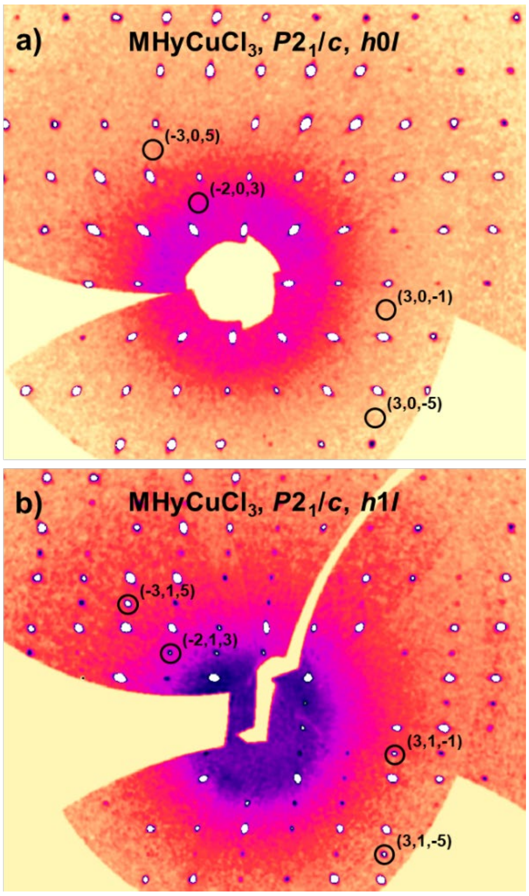

Figure S3. Reciprocal space reconstruction of (a) the *h*0*l* and (b) the *h*1*l* layers in $MHyCuCl_3$; (a) an extinction of reflexes with $l=2n+1$ points out to existence of a *c* glide plane perpendicular to the [010] direction; (b) the analogous reflexes are visible in the *h*1*l* layer. Thus, the unit cell of $P2_1/m$ symmetry and halved *c* axis, as suggested by PLATON software, cannot be chosen.

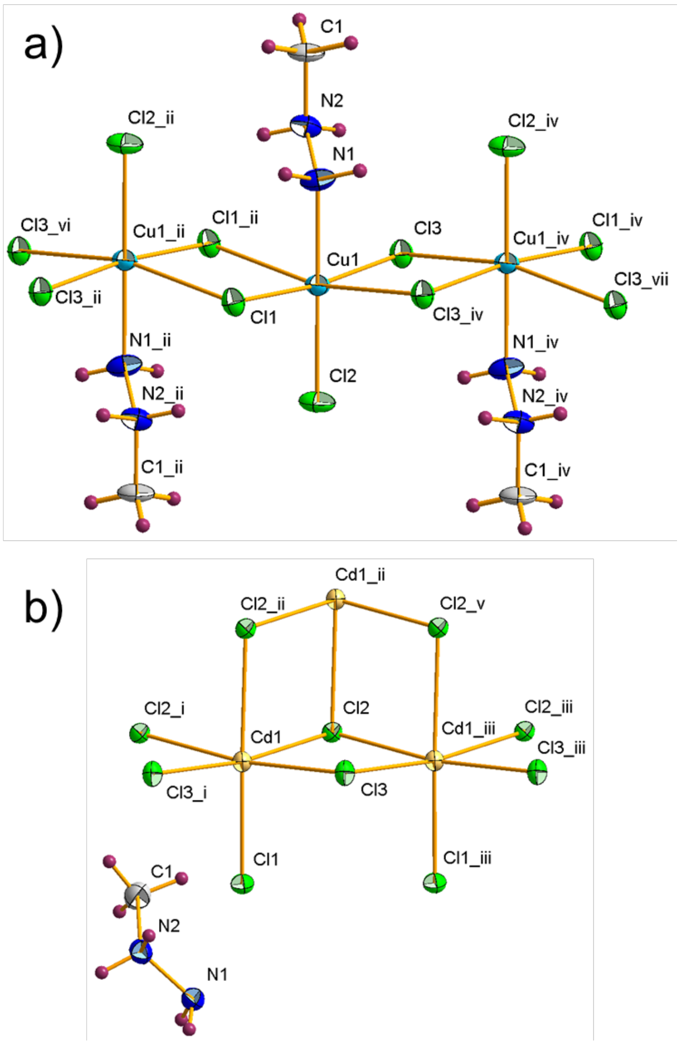

Figure S4. The main components of the crystal structures of a) $MHyCuCl_3$ and b) $MHyCdCl_3$ with vibrational ellipsoids at the 50% probability level. Roman numbers denote symmetry codes: (i) *x*+1, *y*, *z*; (ii) -*x*+1, -*y*+2, -*z*+1; (iii) *x*-1, *y*, *z*; (iv) -*x*+1, -*y*+1, -*z*+1; (v) -*x*, -*y*+2, -*z*+1; (vi) *x*, *y*+1, *z*; (vii) *x*, *y*-1, *z*.

Table S1. Factor group analysis for MHyCdCl$_3$ and MHyCuCl$_3$ crystals.

Ion	Vibrational mode	Free ion symmetry C_s=*m*	Site symmetry [a] C_1=1	Factor group symmetry MHyCdCl$_3$ $C_i=\bar{1}$	Factor group symmetry MHyCuCl$_3$ C_{2h}=2/*m*)
	Internal				
	$\nu_s NH_2+\nu_s NH_2^+$	2A′	2A	$2A_g+2A_u$	$2A_g+2B_g+2A_u+2B_u$
	$\nu_{as} NH_2+\nu_{as} NH_2^+$	2A″	2A	$2A_g+2A_u$	$2A_g+2B_g+2A_u+2B_u$
	$\delta NH_2+\delta NH_2^+$	2A′	2A	$2A_g+2A_u$	$2A_g+2B_g+2A_u+2B_u$
	$\varrho NH_2+\varrho NH_2^+$	2A″	2A	$2A_g+2A_u$	$2A_g+2B_g+2A_u+2B_u$
	$\tau NH_2+\tau NH_2^+$	2A″	2A	$2A_g+2A_u$	$2A_g+2B_g+2A_u+2B_u$
	$\omega NH_2+\omega NH_2^+$	2A′	2A	$2A_g+2A_u$	$2A_g+2B_g+2A_u+2B_u$
	$\nu_s CH_3$	A′	A	A_g+A_u	$A_g+B_g+A_u+B_u$
	$\nu_{as} CH_3$	A′+A″	2A	$2A_g+2A_u$	$2A_g+2B_g+2A_u+2B_u$
MHy^+	$\delta_s CH_3$	A′	A	A_g+A_u	$A_g+B_g+A_u+B_u$
	$\delta_{as} CH_3$	A′+A″	2A	$2A_g+2A_u$	$2A_g+2B_g+2A_u+2B_u$
	ϱCH_3	A′+A″	2A	$2A_g+2A_u$	$2A_g+2B_g+2A_u+2B_u$
	τCH_3	A″	A	A_g+A_u	$A_g+B_g+A_u+B_u$
	ν_{as}CNN	A″	A	A_g+A_u	$A_g+B_g+A_u+B_u$
	δ_sCNN	A′	A	A_g+A_u	$A_g+B_g+A_u+B_u$
	δCNN	A′	A	A_g+A_u	$A_g+B_g+A_u+B_u$
	External				
	T′	2A′+A″	3A	$3A_g+3A_u$	$3A_g+3B_g+3A_u+3B_u$
	L	A′+2A″	3A	$3A_g+3A_u$	$3A_g+3B_g+3A_u+3B_u$
Cl^-	T′		3A	$9A_g+9A_u$	$9A_g+9B_g+9A_u+9B_u$
M^{II}	T′		3A	$3A_g+3A_u$	$3A_g+3B_g+3A_u+3B_u$

Key: [a] the same for both crystals; ν_s, symmetric stretching; ν_{as}, antisymmetric stretching; δ, bending; δ_s, symmetric bending; δ_{as}, antisymmetric bending; ϱ, rocking; τ, twisting; ω, wagging; T′, translation; L, libration; Colors: green, IR- and Raman-active; blue, Raman-active; red, IR-active.

Table S2. Assignment of IR and Raman bands for $MHyCdCl_3$ and $MHyCuCl_3$.

$MHyCdCl_3$		$MHyCuCl_3$		Assignments
**Raman**	**IR (KBr)**	**Raman**	**IR (nujol)**	
3288m	3290m			$\nu_{as}NH_2$
3240m	3255sh, 3241m, 3223sh	3238sh, 3202w	3236m, 3198m	$\nu_{as}NH_2$
3171w	3164sh	3122w	3161m, 3115m	$\nu_{s}NH_2$
3091vw	3117s, 3085s	3077vw		$\nu_{as}NH_2^+$
3043w, 3027m	3029sh	3048sh, 3041m	3049m, 3033m	$\nu_{s}NH_2^+ + \nu_{as}CH_3$
2962vs	2995sh, 2961sh	2967vs	*	$\nu_{s}CH_3$
2886w, 2806w, 2748w	2839vw, 2747w, 2673sh	2908vw, 2807w	*	νNH_2
1606m	1604m	1587w	1596m	δNH_2
1551m	1558m	1566vw	1564s	δNH_2^+
1477sh, 1460m, 1442s	1481w, 1459w, 1449sh, 1443m	1462vw, 1444vw	*	$\delta_{as}CH_3$
1424w, 1413vw	1420vw, 1414w	1431vw, 1410vw	*	$\delta_{s}CH_3$
	1396m		*	ωNH_2^+
1336w	1331vw		*	τNH_2^+
1210m	1233m, 1209w	1278vw, 1252m	1280vw, 1252s	$\rho CH_3 + \omega NH_2$
1133m, 1101m	1134vw, 1100m	1111w	116sh, 1109s	$\varrho NH_2 + \tau NH_2$
1010m	1011m	1020w	1022w	$\nu_{as}CNN$
900vs	901vw	899w	901m	$\nu_{s}CNN$
876s	883m, 875m, 845w	859vw	857m	ϱNH_2^+
389w	386m	614vw	606s	MHy^+-cage
480m	476m	525vw	528vw	δCNN
283m, 253s, 213s	295w, 243s, 209s, 197s	280sh, 271s, 237w	285vs, 241m	$\nu(M^{II}Cl_5N)/\nu(CdCl_6)$+T'(MHy)+L(MHy)
163sh, 150s, 121m	160vs, 135s	187m, 165m, 126m	192s, 146s, 117s	$\delta(CuCl_5N)/\delta(CdCl_6)$+T'(MHy)+L(MHy)
107sh, 87sh, 86vw	106s, 91m, 73m	107s, 85m, 77sh	95sh	$\delta(CuCl_5N)/\delta(CdCl_6)$+T'(MHy)+L(MHy)

Table S3. Experimental and refinement details of $MHyCdCl_3$ and $MHyCuCl_3$.

	**$MHyCdCl_3$**	**$MHyCuCl_3$**
**Crystal data**		
M_r	265.84	216.98
Crystal system, space group	Triclinic, $P\overline{1}$	Monoclinic, $P2_1/c$
Temperature (K)	265	295
a, *b*, *c* (Å)	3.8660 (1), 9.3519 (9), 10.0790 (3)	6.9879 (5), 7.2032 (5), 13.0105 (9)
α, β, γ (°)	106.15 (1), 90.08 (1), 93.73 (1)	90, 96.02 (1), 90
V (Å^3)	349.2 (1)	651.3 (1)
Z	2	4
μ (mm^{-1})	4.16	4.47
Crystal size (mm)	0.15 × 0.06 × 0.05	0.14 × 0.09 × 0.06
**Data collection**		
T_{min}, T_{max}	0.940, 1.000	0.930, 1.000
No. of measured, independent and observed [$I > 2\sigma(I)$] reflections	9262, 2226, 1954	2773, 1237, 979
R_{int}	-	0.023
$(\sin \theta/\lambda)_{max}$ (Å^{-1})	0.610	0.610
**Refinement**		
$R[F^2 > 2\sigma(F^2)]$, $wR(F^2)$, S	0.021, 0.062, 1.08	0.026, 0.064, 1.12
No. of reflections	2226	1237
No. of parameters	68	66
$\Delta\rho_{max}$, $\Delta\rho_{min}$ (e Å^{-3})	0.43, -0.48	0.37, -0.38

Table S4. Selected geometric parameters of $MHyCdCl_3$ and $MHyCuCl_3$ (Å, °).

**$MHyCdCl_3$**		**$MHyCuCl_3$**	
Cd1—Cl2^{i}	2.6953 (11)	Cu1—Cl1^{ii}	2.8149 (9)
Cd1—Cl2	2.7124 (11)	Cu1—Cl1	2.3141 (8)
Cd1—Cl2^{ii}	2.6978 (10)	Cu1—Cl3	2.2990 (8)
Cd1—Cl3	2.6259 (11)	Cu1—Cl3^{iv}	2.7692 (9)
Cd1—Cl3^{i}	2.6235 (11)	Cu1—Cl2	2.2691 (10)
Cd1—Cl1	2.5228 (10)	Cu1—N1	2.061 (3)
Cl2^{ii}—Cd1—Cl2	82.38 (3)	Cl1—Cu1—Cl1^{ii}	89.46 (3)
Cl2^{i}—Cd1—Cl2	91.27 (3)	Cl1—Cu1—Cl3^{iv}	88.95 (3)
Cl2^{i}—Cd1—Cl2^{ii}	84.13 (3)	Cl3—Cu1—Cl1	172.80 (3)
Cl3^{i}—Cd1—Cl2^{i}	86.71 (3)	Cl3—Cu1—Cl1^{ii}	90.03 (3)
Cl3^{i}—Cd1—Cl2^{ii}	90.56 (3)	Cl3^{iv}—Cu1—Cl1^{ii}	166.92 (3)
Cl3—Cd1—Cl2^{i}	172.71 (3)	Cl3—Cu1—Cl3^{iv}	89.93 (3)
Cl3^{i}—Cd1—Cl2	172.82 (3)	Cl2—Cu1—Cl1	92.98 (3)
Cl3—Cd1—Cl2	86.30 (3)	Cl2—Cu1—Cl1^{ii}	96.07 (3)
Cl3—Cd1—Cl2^{ii}	88.73 (3)	Cl2—Cu1—Cl3	94.21 (3)
Cl3^{i}—Cd1—Cl3	94.86 (3)	Cl2—Cu1—Cl3^{iv}	96.98 (3)
Cl1—Cd1—Cl2^{i}	93.53 (3)	N1—Cu1—Cl1	86.47 (8)
Cl1—Cd1—Cl2^{ii}	174.07 (3)	N1—Cu1—Cl1^{ii}	84.58 (8)
Cl1—Cd1—Cl2	92.25 (3)	N1—Cu1—Cl3^{iv}	82.36 (8)
Cl1—Cd1—Cl3^{i}	94.74 (4)	N1—Cu1—Cl3	86.33 (8)
Cl1—Cd1—Cl3	93.44 (3)	N1—Cu1—Cl2	179.15 (8)
Cd1^{iii}—Cl2—Cd1^{ii}	95.87 (3)	Cu1—Cl3—Cu1^{iv}	90.07 (3)
Cd1^{ii}—Cl2—Cd1	97.62 (3)		
Cd1^{iii}—Cl2—Cd1	91.27 (3)		
Cd1^{iii}—Cl3—Cd1	94.86 (3)		

Symmetry code(s): (i) *x*+1, *y*, *z*; (ii) -*x*+1, -*y*+2, -*z*+1; (iii) *x*-1, *y*, *z*; (iv) -*x*+1, -*y*+1, -*z*+1.

Table S5. Selected hydrogen bond parameters of $MHyCdCl_3$ and $MHyCuCl_3$.

D—H···*A*	*D*—H (Å)	H···*A* (Å)	*D*···*A* (Å)	*D*—H···*A* (°)
**$MHyCdCl_3$**				
N2—H2A···Cl1	0.89	2.46	3.168 (4)	137.4
N2—H2B···N1^{i}	0.89	2.10	2.975 (5)	166.6
N1—H1A···Cl3^{ii}	0.89	2.59	3.404 (4)	151.7
N1—H1B···Cl1^{iii}	0.85	2.68	3.384 (4)	140 (4)
**$MHyCuCl_3$**				
N2—H2A···Cl1^{iv}	0.89	2.48	3.157 (3)	132.8
N2—H2B···Cl1^{v}	0.89	2.51	3.269 (3)	143.7
N2—H2B···Cl3	0.89	2.65	3.206 (3)	121.3
N1—H1A···Cl2^{i}	0.89	2.82	3.426 (3)	126.5
N1—H1B···Cl2^{vi}	0.89	2.80	3.631 (3)	155.2

Symmetry code(s): (i) *x*+1, *y*, *z*; (ii) *x*+1, *y*-1, *z*; (iii) -*x*+2, -*y*+1, -*z*; (iv) -*x*+1, -*y*+2, -*z*+1; (v) *x*, -*y*+3/2, *z*-1/2; (vi) -*x*+1, -*y*+1, -*z*+1.
